# Interleukin-6 Derived from the Central Nervous System May Influence the Pathogenesis of Experimental Autoimmune Encephalomyelitis in a Cell-Dependent Manner

**DOI:** 10.3390/cells9020330

**Published:** 2020-01-31

**Authors:** Paula Sanchis, Olaya Fernández-Gayol, Gemma Comes, Anna Escrig, Mercedes Giralt, Richard D. Palmiter, Juan Hidalgo

**Affiliations:** 1 Institute of Neurosciences and Department of Cellular Biology, Physiology and Immunology, Faculty of Biosciences, Universitat Autònoma de Barcelona, 08193 Barcelona, Spain; paula.sanchis@uab.cat (P.S.); of2194@cumc.columbia.edu (O.F.-G.); Annesmon@gmail.com (A.E.); merce.giralt@uab.cat (M.G.); 2 Current affiliation: Department of Pediatrics, Division of Molecular Genetics, Columbia University Irving Medical Center, New York, NY10032, USA; 3 Department of Biochemistry, Genome Sciences, and Howard Hughes Medical Institute, University of Washington, Seattle, WA 98195, USA; palmiter@uw.edu

**Keywords:** conditional IL-6 knockout, experimental autoimmune encephalomyelitis, MOG_35-55_peptide, neuroinflammation

## Abstract

Background: Interleukin-6 (IL-6) is a pleiotropic and multifunctional cytokine that plays a critical role in induction of experimental autoimmune encephalomyelitis (EAE), a mouse model of multiple sclerosis (MS). Although EAE has always been considered a peripherally elicited disease, *Il6* expression exclusively within central nervous system is sufficient to induce EAE development. Neurons, astrocytes, and microglia can secrete and respond to IL-6. Methods: To dissect the relevance of each cell source for establishing EAE, we generated and immunized conditional *Il6* knockout mice for each of these cell types with myelin oligodendrocyte glycoprotein 35-55 (MOG_35-55_) peptide dissolved in complete Freund’s adjuvant (CFA) and supplemented with *Mycobacterium tuberculosis*. Results and conclusions: The combined results reveal a minor role for *Il6* expression in both astrocytes and microglia for symptomatology and neuropathology of EAE, whereas neuronal *Il6* expression was not relevant for the variables analyzed.

## 1. Introduction

Interleukin 6 (IL-6) is a pleiotropic and multifunctional cytokine that has been linked to immune system control and has also been involved in the coordination between endocrine and central nervous systems (CNS) in both basal and inflammatory conditions [1]. Overproduction of IL-6 has been described in several inflammatory and immune disorders such as multiple sclerosis [2,3].

Multiple sclerosis (MS) is the most common disabling autoimmune disease of the CNS in young adults and affects more than 2 millions of people worldwide [4]. Different MS patterns based on imaging and biological markers as well as disease activity and progression have been described. Patients are not usually affected by a single clinically isolated attack, rather many of them develop a relapsing-remitting pattern and suffer progressive disability during the course of the disease. The major pathological features of MS are demyelinating lesions in white matter of the CNS accompanied by lymphocyte infiltrates, high production of cytokines, and gliosis, which cause neuronal damage [5]. Although the etiology of MS is unknown, a complex interaction among genetic, environmental, and lifestyle factors could contribute to disease susceptibility [6]. Increased levels of *Il6* mRNA and/or IL-6 protein have been detected in serum, cerebrospinal fluid, and nervous tissue from MS patients [3,7,8]. Moreover, polymorphisms of the *Il6* gene could are associated with higher MS risk [9,10,11]. Different experimental models have been used to mimic features of MS disease. Experimental autoimmune encephalomyelitis (EAE) produced by active immunization is one of the most used models to study acute and chronic inflammation in the spinal cord [12]. This model consists in the injection of the Myelin oligodendrocyte glycoprotein 35-55 (MOG_35-55_) peptide dissolved in complete Freund’s adjuvant (CFA) and supplemented with *Mycobacterium tuberculosis* [13], which causes an inflammatory encephalomyelitis with primary axonal injury mediated by T-helper cells producing IL-17 (Th17) [14]. IL-6 is a critical cytokine in the induction phase of EAE pathogenesis, as total *Il6*-deficient mice (IL-6 KO) are resistant to EAE and IL-6 blockade inhibits the development of EAE symptoms [15,16,17,18,19,20]. Several mechanisms may underlie this resistance. For instance, IL-6 KO mice showed an incompetent Th17 response and presented few infiltrates of T lymphocytes and monocytes [17,21]. Indeed, it has been described that IL-6 blocks TGFβ-induced regulatory T cells (Treg) differentiation and induces, together with TGFβ, the retinoid-related orphan-γt and -α receptors (ROR-γt and ROR-α), which are key transcription factors for Th17 differentiation [21,22,23,24]. IL-6 has also been involved in the regulation of adhesion molecules in endothelial cells, thus regulating the trafficking of infiltrating cells [17]. However, the role of IL-6 in MS and EAE dynamics is unclear, given that, surprisingly, IL-6 blockade is ineffective once EAE starts [19]. Moreover, some authors have observed a neuroprotective role of IL-6 since it might induce myelin repair in inactive demyelinated lesions from MS patients [7] or even that the exogenous administration of IL-6 or its production may reduce the clinical signs of EAE [25,26,27]. With respect to the production of IL-6 by peripheral cells, it is known that IL-6 from T cells, B cells, and dendritic cells promotes the clinical signs of EAE [28,29]. However, only dendritic cell-derived *Il6* deficiency was able to completely block the EAE induction [29].

IL-6 can be produced within the CNS in neuroinflammatory conditions by a wide variety of brain cells and infiltrating inflammatory cells. Thus, studying the multiple cellular sources of IL-6 could help to better understand the contribution of each one to the pathological process of EAE. Astrocytes have been involved in each stage of the EAE pathologic process from the scar-like formation to restrict the inflammation to the axonal damage before EAE symptoms begin [30,31]. Astrocytes are one of the major sources of IL-6 in EAE and its production has been shown to be increased in areas of injury by proximal astrocytes [3,32,33], and in a preliminary study we showed that MOG_35-55_-immunized astrocytic *Il6*-deficient females had a delay and an amelioration of EAE clinical signs [34]. Nevertheless, some evidence that IL-6 secreted by astrocytes could be detrimental is also available, since mice with a chronic non-resolving form of EAE displayed IL-6 production mainly in astrocytes, and when they were injected with the IL-6 antibody, their clinical signs improved [33]. These authors suggested that the detrimental role of astrocyte-derived IL-6 during EAE might be mediated through microglia, since an upregulation of *Galectin-1* expression was observed in the brain, which is secreted by astrocytes and limits microglial activation [33]. Microglia are resident immune cells of the CNS that can acquire a phagocytic phenotype. Microglia are resident immune cells of the CNS that can acquire a phagocytic phenotype. Besides astrocytes, microglia are one of the main sources and target of IL-6 following neuroinflammatory processes [35]. Although most data suggest that microglial activation begins after formation of demyelinating MS plaques, in some cases their activation has been described before demyelinating processes and axonal injuries [36]. Different roles for their activation have been reported in MS and EAE pathogenesis, including myelin debris removal, antigen presentation, and cytokine production. Moreover, other study also reported that inhibiting microglia activation would delay and reduce the EAE clinical score [37]. Recent data have highlighted the possible neuroprotective effects of IL-6 derived from microglia and infiltrated macrophages in EAE pathogenesis [29,38]. Another cellular source of IL-6 within the CNS is neurons [39,40]; however, the way in which neuronal IL-6 participates in EAE pathogenesis has not yet been studied.

Since EAE pathogenesis is coordinated by IL-6 and brain production of IL-6 could be relevant in the symptomatology and inflammatory response of this disease, a thorough understanding of role of IL-6 derived from astrocytes, neurons, and microglia is important. In this study, we have investigated the role or astrocytes, microglia, and neurons in the MOG_35-55_-immunization-induced EAE. Our results suggest that astrocytic and microglial IL-6, but not neuronal IL-6, play a role in EAE symptomatology and the subsequent neuropathology in a sex-dependent manner.

## 2. Materials and Methods

### 2.1. Animals

All mice used in this study were fed ad libitum and housed under constant temperature and 12h light/ 12h dark cycle. All experiments were performed with approval by the Ethics Committee on Animal Experiments of the Universitat Autònoma de Barcelona and the Generalitat de Catalunya (Refs. 3782 and 9684, respectively).

#### Conditional IL-6 KO Mice

To obtain conditional KO mice where *Il6* expression was blunted in astrocytes, neurons, or microglia, we used *Il6*^lox/lox^ mice [41] and mice in which Cre recombinase was driven by Gfap [42], Syn1 [43], or Cx3cr1 [44] promoters, respectively. *Il6*^lox/lox^ mice were back-crossed C57BL/6OlaHsd mice for more than 10 generations before being crossed with Cre-expressing mice. The offspring positive for Cre were selected and crossed again with *Il6*^lox/lox^ mice obtaining four genotypes: *Il6*^lox/wt^ Cre^+/-^, *Il6*^lox/wt^ Cre^-/-^, *Il6*^lox/lox^ Cre^+/-^, and *Il6*^lox/lox^ Cre^-/-^. For the different experiments, we selected as subjects of study the *Il6*^lox/lox^ Cre^+/-^ (IL6:GFAP KO, IL6:SYN1 KO, or IL6:CX3CR1 KO), which lacked *Il6* expression in astrocytes, neurons, or microglia, respectively, and as controls the *Il6*^lox/lox^ Cre^-/-^ (*Il6*^lox/lox^ (floxed) mice, which is wild type (WT)).

### 2.2. Genotyping

All mice were genotyped by PCR analysis of tail, liver, and brain DNA, which were previously extracted by boiling in 100 µL of 50 mM sodium hydroxide for 7 min. This is essential to exclude animals with ectopic recombination. The primers and PCR protocol to genotype the *Il6* gene and *Cre recombinase* of the conditional IL-6 KO mice were obtained from Quintana et al. [41] and Sanz et al. [45].

### 2.3. Animals Used for EAE and Tamoxifen Treatment

The induction of EAE was carried out using adult mice. A single experiment was carried out with IL6:GFAP KO and their WT mice (2–3 months old); and with IL6:CX3CR1 KO and their WT mice (3–5 months old). Because of the very high rate of ectopic recombination observed (which severely restricted the number of mice usable for experiments; see below), two separate experiments were carried out with IL6:SYN1 KO and WT mice, which have been pooled; most of the mice from both experiments were 4–11 months old, and 1 male KO and 1 female WT were 2 months old. Gfap-Cre and Synapsin-1-Cre mice are constitutive models [42,43], whereas Cx3cr1^CreER^ mouse is a tamoxifen-inducible model [44]. Therefore, the IL6:CX3CR1 KO mice and their respective controls were injected with Tamoxifen (TAM). TAM solution was prepared in two steps: (i) First, TAM (Sigma 5648, Madrid, Spain) was dissolved in ethanol at a concentration of 10 mg/100 µL by shaking and heating at 37 °C [46], and then, (ii) the solution was diluted with sunflower oil to 10 mg/mL. Mice received one intraperitoneal injection of 100 µL of solution (1 mg of TAM) per day for five consecutive days, and they were used for EAE experiments 8 weeks later.

### 2.4. Induction of EAE and Clinical Evaluation

The induction of EAE was carried out as follows. Mice were anesthetized by inhalation with isoflurane (oxygen flowmeter to 0.8 L/min and isoflurane vaporizer to 4% and 1.5% during induction and maintenance, respectively) as described [13,15]. The emulsion was prepared by sonication from two separate solutions in equal volumes: one in which the lyophilized MOG_35–55_ peptide (MEVGWYRSPFSRVVHLYRNGK, purity >98%; peptide synthetized under request in the Peptide Synthesis Facility at the Universitat Pompeu Fabra, Barcelona, Spain) was diluted with 10 mM phosphate buffered saline (PBS 1X) to 3 mg/mL, and another in which the *M. tuberculosis* H37RA (BD Difco 231141, New Jersey, USA) was diluted with complete Freund’s adjuvant (CFA; Sigma-Aldrich F5881, St. Louis, MO, USA) to 4 mg/mL. On day 0, mice were immunized with two subcutaneous injections of 100 µL of MOG_35–55_ emulsion into the hind flanks, whereas those used as controls (whenever possible) were immunized instead with an emulsion containing 3 mg/mL of bovine serum albumin (BSA; Sigma A9647). Additionally, mice were injected intraperitoneally with 2.5 μg/mL *Bordetella pertussis* toxin (Native Antigen Company PT-TNL-50, UK) to facilitate the EAE induction. A second dose of *B. pertussis* toxin was injected 2 days after immunization.

Body weight and progression of EAE were monitored daily after immunization, with the latter being assessed in each animal with a numerical scale following criteria: 0 = no signs of disease, 0.5 = partial loss of tail tonus, 1 = loss of tail tonus, 2 = moderate hind limb paraparesis, 2.5 = severe hind limb paraparesis, 3 = partial hind limb paralysis, 3.5 = hind limb paralysis, 4 = tetraplegia, and 5 = death. When mice lost more than 10% weight, they were injected with saline supplemented with 3.6% glucose. The self-mutilation, weight loss greater than 25%, and tetraplegia for more than two days were considered as endpoints. The time to disease onset, time to peak disease, peak-score, and cumulative score (sum of all scores) were also calculated individually [13,15].

Mice were killed by decapitation and the blood was collected from the trunk, with the serum obtained by centrifugation at 9500× *g* for 10 min at 4 °C and stored at −85 °C. The vertebral column was cut between the ninth and tenth thoracic vertebrae, and the upper part was fixed with 4% paraformaldehyde (PFA) for histological analysis, while the lower part was carefully dissected, snap-frozen in liquid nitrogen, and stored at −80 °C for molecular analysis. In the case of IL6:GFAP KO and their controls, the medulla oblongata was used for molecular analysis and the entire spinal cord was fixed for histological analysis.

### 2.5. Quantitative Polymerase Chain Reaction (qPCR)

RNA was extracted from spinal cord (~20 mg) of BSA- and MOG_35-55_-immunized mice using Promega Maxwell RSC simplyRNA kit following manufacturer’s instructions. Before synthesizing cDNA using iScript cDNA synthesis kit (BioRad Laboratories, Madrid, Spain), 2 µg of RNA of each sample was treated with an extra DNase I step (Qiagen, Hilden, Germany). Then, a two-step qPCR protocol was performed using the iTaq Universal SYBR Green Super-mix (1725124, BioRad Laboratories, Madrid, Spain) and different pairs of primers. Samples were loaded in duplicate to the plate at the appropriate concentration, which depended on the pair of primers (Supplementary Figure S1). In addition, primers for *Glyceraldehyde 3-phosphate dehydrogenase* (*Gapdh*) gene were used in each cDNA concentration and a 3-point 10-fold dilution curve (sample mixture) was also included to obtain the efficiency of amplification of the primers of interest.

The analysis of fold change expression was calculated using the delta-delta-Ct method with different efficiencies, which were obtained from the slope of the standard curve for each gene, and *Gapdh* as reference gene. Finally, the average of whole BSA-immunized WT male group was used as calibrator in the experiment of gene expression of IL6:CX3CR1 KO mice and WT mice, and its expression change average was always 1. In the other gene expression experiments (IL6:GFAP KO mice and IL6:SYN1 KO their respective controls), the calibrator was the whole MOG_35-55_-immunized WT male group.

### 2.6. Histology

The vertebral column with spinal cord was carefully dissected, removing the vertebral column and post-fixing the spinal cord in fresh 4% PFA for 24 h. Afterwards, the spinal cord was washed with PBS 1X, embedded in paraffin and cut sagittally into 8-μm wide sections. For myelin evaluation, samples were incubated with 0.1% Luxol Fast Blue (LFB) overnight at 56 °C and then differentiated with 0.05% lithium carbonate (Fluka 62K70, Bucharest, Romania) and 70% ethanol. Samples were counterstained with hematoxylin solution (Sigma MHS16, Madrid, Spain). Other sections were used for IHC analysis, with microglia/macrophage, astrocytes, lymphocytes cluster of differentiation 3 + (CD3+), and Th17 being identified by antibodies against ionized calcium binding adaptor molecule-1 (IBA-1; Wako Pure Chemical industries 019-19741, 1/1500, Osaka, Japan), GFAP (Dako Z0334, 1/900, Denmark A/S), CD3+ (Dako A0452, 1/100, Denmark A/S), and IL-17 (Santa Cruz Biotechnology 7927, 1/50, Heidelberg, Germany), respectively. Endothelial cells were detected by antisera against Factor Von Willebrand (FVW; Dako A0082, 1/800, Denmark A/S).

Antigen retrieval treatment was required in IBA-1, CD3, IL-17, and FVW IHC. For the former, samples were treated with citrate buffer (10 mM sodium citrate 0.05% tween, pH 6) for 20 min at 96 °C whereas for the latter three, sections were treated with 1 g/L protease type XIV (Sigma, P5147, Madrid, Spain) for 8 min at 37 °C. Then, blocking endogenous peroxidase was performed using 70% methanol and 3% hydrogen peroxide (H2O2) for 15 min. Afterwards, samples were blocked in 1% BSA in Tris-buffered saline 0.05 M and 0.5% triton at least for 1 h and then incubated with primary antibodies overnight at 4 °C. The following day, samples were incubated with secondary antibody Atom BA-1000 (biotininylated anti-rabbit IgG (H + L) 1/300, Vector Laboratories, Burlingame, CA, USA) for 1 h at room temperature (RT) and later with horseradish peroxidase-coupled streptavidin (Vector SA-5004, 1/500-600, Burlingame, CA, USA). Immunoreactivity was visualized by adding 50 mg/mL 3,3-diaminobenzidine-tetrahydrohloride and 0.033% H_2_O_2_ for 1–5 min at RT. Finally, slides from CD3 and FVW IHC were counterstained with hematoxylin solution.

Two non-consecutive sections from each animal were used for each histological analysis. Serial images of the tissues from duplicates were taken at 10-20x using a Nikon Eclipse 90i microscope and Nikon Act-1 software. Samples were quantified using ImageJ software, and in those of CD3 and FVW IHQ counterstained with hematoxylin and of LFB/hematoxylin staining, we previously used the color deconvolution plugin of Image J to divide both colors and quantify only CD3+ cells, vessels, and myelin. In LFB staining and IL-17 IHC, the percentage of demyelinated and infiltrated area, respectively, was evaluated in the white matter. For CD3 IHC, we quantified the integrated density of CD3 per infiltrate. For IBA-1 and GFAP IHC, quantification of integrated density of white and grey matter of the spinal cord was annotated. This was not possible in the case of IBA-1 staining of the white matter of IL6:GFAP KO cross because background was too high; therefore, we counted instead the reactive IBA-1+ cells per infiltrate. For FVW IHC, we focused on integrated density of white matter. All results were normalized using all the measured area.

### 2.7. Statistics

All data are presented as mean ± SEM. Statistical calculations were performed using the Statistical Package for Social Sciences (SPSS) software. The clinical score was statistically analyzed by generalized linear model (GzLM) using the area under curve measure, which was previously calculated using GraphPad Prism. Body weight gain and the other clinical evaluations were analyzed by generalized estimated equations (GEE) and Mann–Whitney U test, respectively. Histological and gene expression analysis were analyzed by GzLM using genotype and sex or genotype and treatment as mean factors. In case of comparisons between two groups, t-Student was used. Statistical significance was set at p ≤ 0.05 in all analyses.

## 3. Results

### 3.1. IL6:GFAP KO, but not IL6:SYN1 KO and IL6:CX3CR1 KO Mice, Present an Ameliorated EAE Symptomatology in a Sex-Dependent Manner

It is increasingly clear that ectopic recombination is widespread among Cre driver lines, which must be properly controlled as thoroughly discussed recently [47]. Therefore, in all mice we assessed specific and nonspecific *Il6* recombination in DNA isolated from brain and tail and liver samples, respectively. In accordance to previous results using GFAP-Cre, Syn1-Cre, and Cx3cr1^CreER^ mice [40,48], ectopic *Il6* recombination (presumably indicating germline recombination) was very high in the former two (~60–90%, respectively) and nearly absent in the latter. Therefore, we only used mice that showed no peripheral recombination, which severely restricted the number of groups possible. MOG_35–55_ peptide and BSA immunizations were possible in the microglial model, but in the case of the astrocytic and neuronal models, prioritizing MOG_35–55_ immunization was the most logical option, and therefore no BSA-immunized animals were employed. Some non-immunized control animals were also included in the astrocytic model for illustrating the effect of EAE on neuropathology in that cross; unfortunately, that was not possible in the neuronal model.

**Figure 1:**
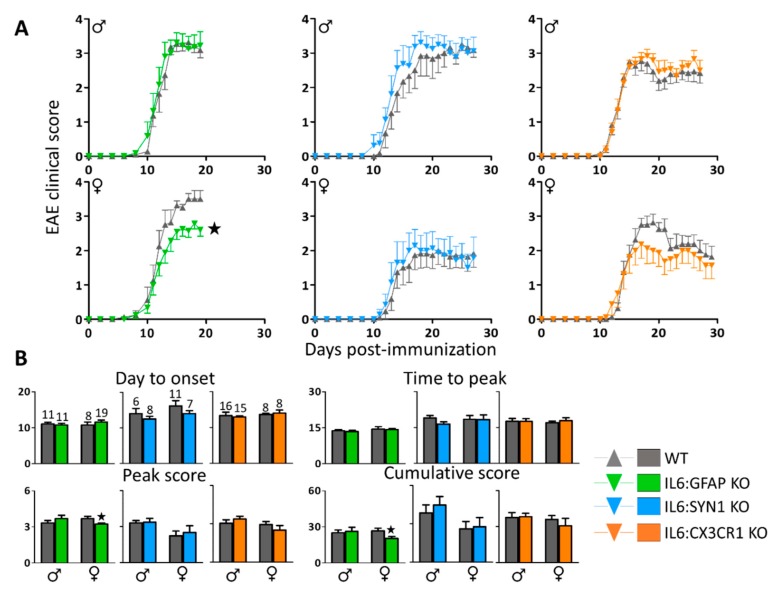
Clinical score after myelin oligodendrocyte glycoprotein 35–55 (MOG_35–55_) immunization separated by sex and genotype. (**A**) Clinical course of EAE of IL6:GFAP KO and wild type (WT) mice (*left*); IL6:SYN1 KO and WT mice (*middle*); IL6:CX3CR1 KO and WT mice (*right*). All MOG_35-55_-immunized mice displayed the prototypical ascending paralysis course starting at 10 dpi, but only IL6:GFAP KO females showed an ameliorated experimental autoimmune encephalomyelitis (EAE) symptomatology. (**B**) For each animal, we determined the time to disease onset, time to peak disease, peak score, and cumulative score (sum of all scores from disease onset to last day annotated). Only IL6:GFAP KO females showed a decrease in peak score and cumulative score. Number of mice per group as indicated. Results are MEAN ± SEM; ★ *p* ≤ 0.05 versus WT mice.

**Figure 2:**
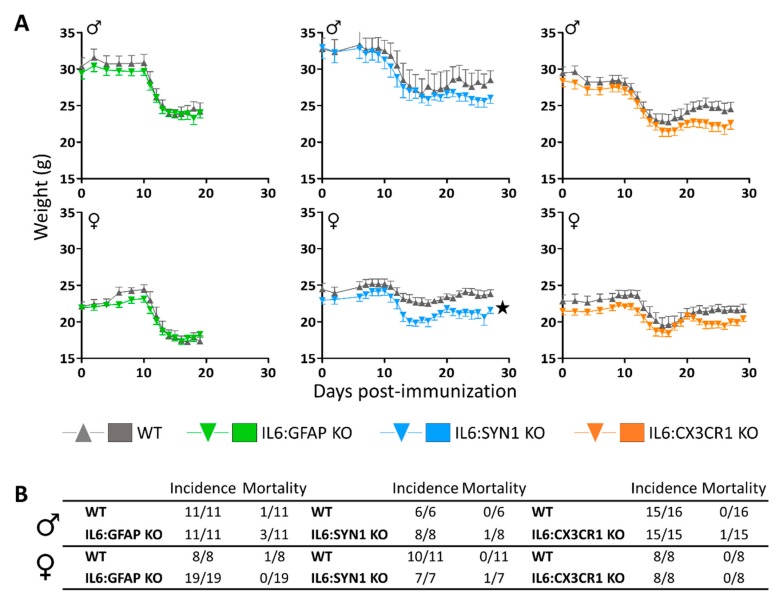
Clinical assessment of EAE. (**A**) Absolute body weight changes of IL6:GFAP KO and WT mice (*left*); IL6:SYN1 KO and WT mice (*middle*); IL6:CX3CR1 KO and WT mice (*right*). All MOG_35-55_-immunized mice displayed a prominent body weight loss peaking approximately at 15–17 dpi. IL6:SYN1 KO females weighed less than their controls regardless of EAE. (**B**) EAE incidence and mortality. Although all mice displayed paralyzing symptoms, the mortality rate was low during the studied days. Number of mice per group as indicated. Results are MEAN ± SEM; ★ *p* ≤ 0.05 versus WT mice.

All MOG_35-55_-immunized mice showed the prototypical ascending paralysis course in the three crosses, starting at 10 days post-immunization (dpi) and peaking approximately at 15-17 dpi with a clinical score of 2-3 (Figure 1A). It is important to emphasize that these are independent experiments, using different Cre-expressing strains that carry different genetic backgrounds, performed not in parallel but using different MOG_35-55_ batches; therefore, they cannot be compared directly with each other. Regarding the development of EAE in mice presumably with (see below) *Il6* expression blunted in astrocytes, microglia, or neurons, there was a clear sex-dependent effect. Thus, no differences were observed in male mice of the three models compared to their respective WT animals. In contrast, female mice lacking IL-6 in astrocytes had a significantly ameliorated clinical EAE score (Figure 1A, *left*), and the same trend was observed in those lacking IL-6 in microglia during the acute phase of the disease (17-21 dpi; P=0.079) (Figure 1A, *right*), whereas no differences were observed in the neuronal model (Figure 1A, *middle*). The day of onset, peak score, time to peak, and cumulative score were also determined for each animal (Figure 1B). In accordance with the previous results, significant changes were observed only in females lacking IL-6 in astrocytes, which showed a decrease in peak score and cumulative score. Although the incidence of EAE was almost 100% for all of the groups, the mortality rate was between 2 and 10 % depending on the cross, with no obvious differences between genotypes (Figure 2B).

In accordance with the clinical scores observed, all EAE mice lost body weight, both absolute (Figure 2A) and relative (Supplementary Figure S2A). Despite the significantly ameliorated clinical EAE score of female mice lacking IL-6 in astrocytes, they lost the same amount of absolute and relative body weight as their WT mice (Figure 2A, *left*; Supplementary Figure S2A, *left*). IL6:SYN1 KO and IL6:CX3CR1 KO male and female mice lost weight similar to their respective WT group (Figure 2A and Supplementary Figure S2A, *middle* and *right,* respectively). Interestingly, females lacking IL-6 in neurons weighed significantly less than their WT throughout the experiment regardless of EAE (Figure 2A, *middle*).

In contrast to MOG_35–55_ immunization, BSA-immunized IL6:CX3CR1 KO and WT mice neither displayed any type of paralyzing symptoms during the 27–29 dpi, nor showed these prominent weight losses (Supplementary Figure S2B,C).

### 3.2. Demyelination, Infiltrates, and Gliosis in the Spinal Cord of MOG_35-55_-Immunized Mice are Regulated in Sex- and Cellular IL-6 Source-Dependent Manner

**Figure 3:**
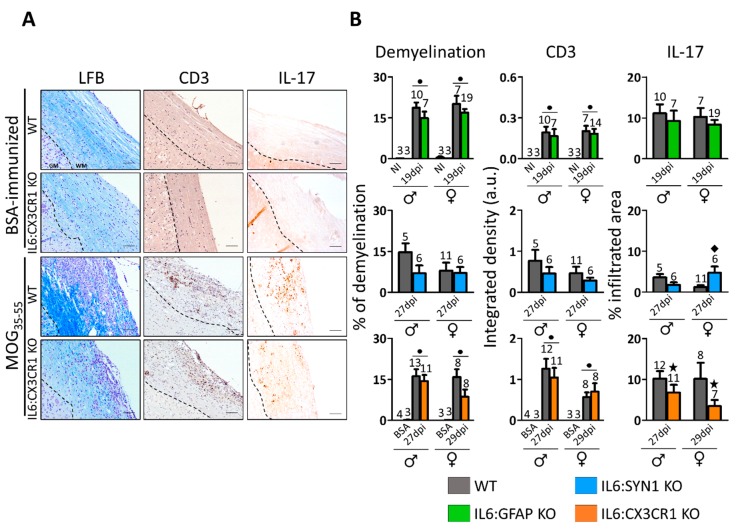
Analysis of demyelination and infiltrates in the white matter of CNS. (**A**) Representative images of LFB staining counterstained with hematoxylin (*left*), CD3 immunostaining counterstained with hematoxylin (*middle*), and Interleukin-17 (IL-17) (*right*) immunostaining of bovine serum albumin (BSA)- and MOG_35–55_-immunized mice. The discontinuous line delimits white matter (WM) from gray matter (GM). MOG_35–55_ immunization caused clear signs of demyelination and prominent accumulation of inflammatory cellular infiltrates. Scale bar: 100 µm. (**B**) Quantification of LFB staining (*left*), CD3+ (*middle*), and IL-17+ (*right*). BSA- and non-immunized (NI) mice had no demyelination or infiltrates in comparison with MOG_35–55_-imunized animals. No significant differences between genotypes were observed other than the decrease of IL-17 IHC in IL6:CX3CR1 KO mice and a sex-dependent opposed effect in IL6:SYN1 KO mice. All results were normalized per total area and represented in MEAN ± SEM. Number of mice per group as indicated. ● *p* ≤ 0.05 versus BSA/NI mice ★ *p* ≤ 0.05 versus WT mice. ◆ Indicates a significant interaction between genotype and sex.

**Figure 4:**
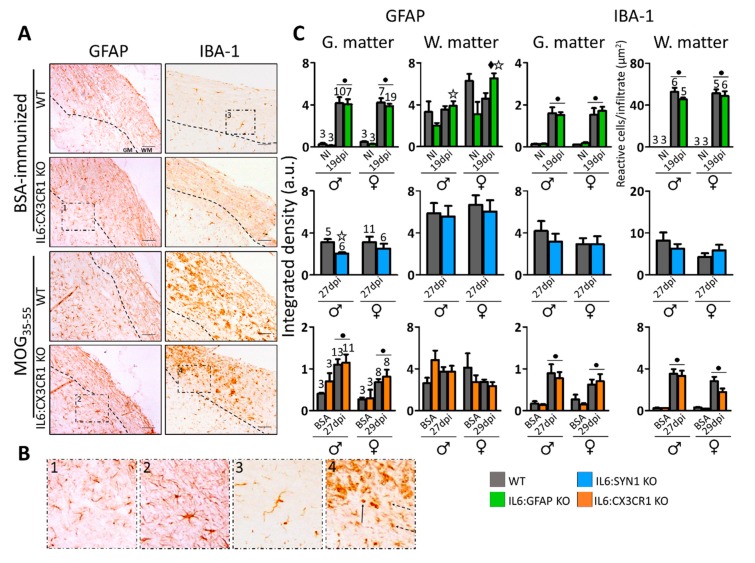
Assessment of the gliosis. (**A**) Representative images of GFAP immunostaining (astrocytes; *left*) and IBA-1 immunostaining (microglia; *right*) of BSA- and MOG_35–55_-immunized IL6:CX3CR1 KO mice. The discontinuous line delimits white matter (WM) from gray matter (GM). MOG_35-55_ immunization caused a dramatic accumulation of activated astrocytes and microglia widespread across white and gray matter of the CNS. Scale bar: 100 µm. (**B**) High magnifications of (A) showing the different morphologies of GFAP+ and IBA-1+ cells. (1) Ramified, resting GFAP+ cells; (2) reactive, hypertrophic GFAP+ cells; (3) ramified, resting IBA-1+ cells; (4) reactive, with shorter and thicker cell processes (dashed black arrows) and round (black arrow) IBA-1+ cells. (**C**) Quantification of GFAP (*left*) and IBA-1 (*right*) IHC in the three crosses revealed activation of astrocytes and microglia, respectively. In general, the effect of EAE was more robust on microgliosis (clearly seen in both white and gray matter areas of the spinal cord) than on astrogliosis (more obvious in gray matter); but there were no clear differences between genotypes in comparison to their control mice. Number of mice per group as indicated. All results were normalized per total area and were represented in MEAN ± SEM; ● *p* ≤ 0.05 versus BSA/NI mice. ◆ Indicates a significant interaction between factors. ✩ *p* ≤ 0.05 versus WT mice in a specific sex of EAE animals.

Representative images of demyelination and CD3+ and IL-17 infiltrates in the spinal cord are shown in Figure 3A and quantification of the results is shown in Figure 3B. In accordance with the clinical signs, the spinal cord of MOG_35–55_-immunized mice, but not those immunized with BSA, showed clear signs of focal demyelination of the white matter and the presence of infiltrates. Quantification of luxol staining showed a general trend for a lower demyelinating area in all the KO mice (Figure 3B, *left*). Using sex as an additional factor in the statistical analysis, astrocytic *Il6* deficiency tended to decrease the percentage of demyelinated area (*p* = 0.075) (Supplementary Figure S3). No effect on the CD3+ infiltrates between genotypes was observed (Figure 3B, *middle*; Supplementary Figure S3; Figure 4). In contrast, there is an interaction between sex and genotype (IL6:SYN1 KO and WT mice) with regard to IL-17 infiltrates and a significant difference between IL6:CX3CR1 KO and WT mice, with IL-17 infiltrates being decreased by lack of microglial IL-6 (Figure 3B, *right*; and Supplementary Figure S4).

Gliosis was evaluated by GFAP (astrocytes) and IBA-1 (microglia) IHC (Figure 4). We observed GFAP+ and IBA-1+ cells widespread across white and gray matter of the spinal cord in MOG_35–55_-immunized IL6:CX3CR1 KO and WT mice compared to those immunized with BSA (Figure 4A,B). Moreover, BSA-immunized IL6:CX3CR1 KO mice showed exclusively GFAP+ and IBA-1+ cells with resting, ramified morphology both in the white and grey matter of spinal cord (Figure 4B, panels 1 and 3). In contrast, after MOG_35-55_-immunization, mice displayed reactive, thicker cell processes, GFAP+ cells, and reactive, less ramified and even round IBA-1+ cells (Figure 4B, panels 2 and 4).

Quantification of GFAP IHC is shown in Figure 4C, *left*. GFAP quantification in the white matter is challenging because background is high, which we think is rather due to myelin tracts than to unspecific staining, since when primary antibody was omitted, the staining was very low (not shown). Therefore, quantification of overall staining intensity did not reveal clear effects of EAE or IL-6 deficiencies in this spinal cord area. In contrast, quantification of GFAP IHC was more meaningful in the gray area, where we could observe clear increases in EAE animals in IL6:GFAP KO mice (compared to non-immunized animals) and in IL6:CX3CR1 KO mice (compared to BSA-immunized animals). The effect of EAE was clearly higher in the former than in the latter cross, perhaps because the timings were different: 19 dpi vs 27–29 dpi, respectively. No clear differences between genotypes were observed when using sex as a factor in the statistical analysis. However, if males and females were analyzed separately, in the IL6:SYN1 KO cross, a significant decrease was observed in the gray matter of IL6:SYN1 KO male mice compared to their WT animals (Figure 4C, *left*).

Quantification of IBA-1 IHC (integrated density) is shown in Figure 4C, *right*. In general, this could be done in both the white and the gray matter, but in the IL6:GFAP KO cross, the background was too high in the white matter (Supplementary Figure S3); therefore, in this case, we instead counted the number of reactive IBA-1+ cells per infiltrate. Regardless of the method employed, the effect of EAE was clearly visible in both areas of the spinal cord when compared with NI or BSA-immunized animals, but again no clear differences between genotypes were observed, other than a trend for decreasing microgliosis in IL6:CX3CR1 KO females (*p* = 0.070) (Figure 4C, *right*). Finally, angiogenesis was analyzed with FVW IHC in the white matter of IL6:GFAP KO and WT mice (Supplementary Figure S5A). This staining was increased in MOG_35-55_-immunized mice compared to NI mice. If the latter are excluded as a factor in the statistical analysis, a significant difference was observed between genotypes (Supplementary Figure S5B).

### 3.3. IL6:GFAP KO and IL6:CX3CR1 KO, but not IL6:SYN1 KO Mice, Present an Altered Expression of Some Inflammation-Related Genes During EAE

**Figure 5:**
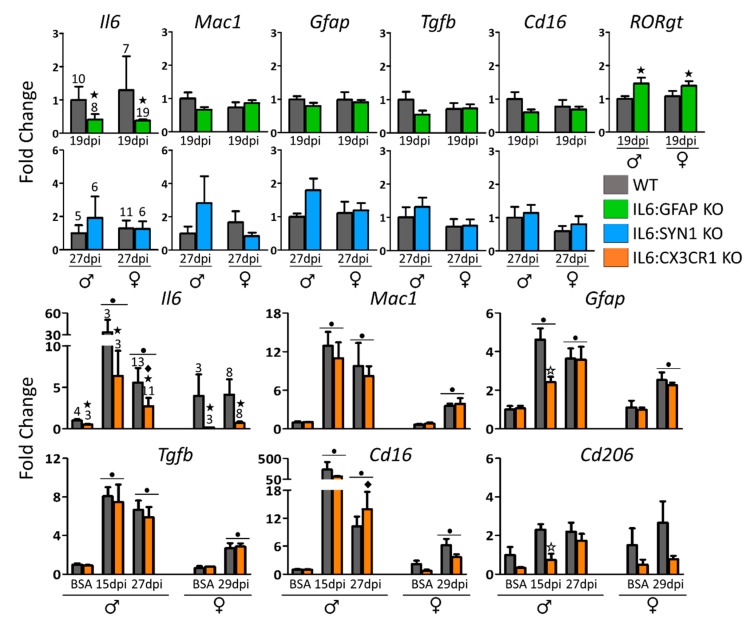
Analysis of inflammation-related gene expression. MOG_35-55_-immunized mice consistently presented with an increased expression of many of the genes studied both at 15 and 27–29 dpi (*bottom*). The *Il6* expression levels were not as higher in MOG_35-55_-immunized IL6:GFAP KO and IL6:CX3CR1 KO mice as their MOG_35-55_-immunized controls. In contrast, they were similar between IL6:SYN1 KO and their control mice. Moreover, astrocytic (*top*), neuronal (*middle*), and microglial (*bottom*) *Il6* deficiency plays a marginal and complex role in the regulation gliosis (*Mac1* and *Gfap* expression) and macrophage polarization profile (*Cd16*, *Cd206,* and *Tgfβ* expression). Number of mice per group as indicated. All results were represented in MEAN ± SEM; ★ *p* ≤ 0.05 versus WT mice. ✩ *p* ≤ 0.05 versus WT mice at 15 dpi only. ● *p* ≤ 0.05 versus BSA-immunized mice. ◆ Indicates a significant interaction between factors.

MOG_35-55_-immunization caused an upregulation of *Il6* mRNA levels of the spinal cord compared to BSA-immunized animals, with the increase being higher at 15 dpi than at 27–29 dpi (Figure 5). At the time periods studied, *Il6* mRNA levels were lower in IL6:GFAP KO and IL6:CX3CR1 KO, but not in IL6:SYN1 KO mice, compared to their respective WT controls.

In addition, to dissect the putative role of IL-6 derived from each cellular source in EAE dynamics, we also analyzed the expression of several genes related to gliosis (*Mac1* and *Gfap*), and M1 (*Cd16*; pro-inflammatory role) and M2 (*Tgfb* and *Cd206*; immunoregulatory role) phenotypic markers in IL6:GFAP KO, IL6:SYN1 KO, and IL6:CX3CR1 KO mice (Figure 5). Concerning the latter model, where we could analyze BSA- and MOG_35-55_-immunized animals, all the genes analyzed but *Cd206* were significantly upregulated during EAE compared to BSA-immunized mice (Figure 5, *bottom*). Microglial *Il6* deficiency tended to decrease some of these responses when analyzing all the groups simultaneously (separately for each sex). If analyzed separately at 15 and 27 dpi, a significant decrease was observed at 15 dpi for *Gfap* and *Cd206* expression in males.

Regarding IL6:GFAP KO and IL6:SYN1 KO mice, we could not include BSA or non-immunized animals (see above). Therefore, the focus is in the putative differences compared to WT animals during EAE. While some trends were observed in astrocytic and neuronal models at 19 and 27 dpi, respectively, the only significant difference was for *RORgt* mRNA levels, which were significantly upregulated in MOG_35-55_-immunized IL6:GFAP KO mice compared to their controls (Figure 5, *top*).

## 4. Discussion

MS is one of first causes of chronic disability among young people worldwide but its underlying pathological mechanisms are still not well defined. In the past years, many groups have described IL-6 as a key mediator at the induction phase of EAE [15,16,17,18,19,20]. This result together with others that demonstrate the possible neuroprotective roles of IL-6 in EAE reflect the multifunctional character of this cytokine [25,26]. Since IL-6 is produced by diverse brain and peripheral cellular types, the study of IL-6 from different cellular sources in the inflammatory response to EAE could give some insight into the heterogeneity of its functions. Although EAE has always been considered as a peripheral disease [49], we observed that *Il6* expression exclusively within CNS could induce the atypical EAE-related symptoms known to occur in GFAP-IL6 mice without systemic IL-6 [15]. This is in agreement with results in systemic IL-6 KO mice reporting that IL-6 could mediate locally EAE disease, since the resistant phenotype cannot be explained solely by lack of encephalitogenic T cells [50]. To dissect the specific role of astrocyte-, neuron-, and microglia-derived IL-6, we have used the Cre-lox system. As stated above, ectopic recombination is widespread among Cre driver lines [47], and therefore ectopic *Il6* recombination was adequately controlled and only animals with the specific recombination were used.

The production of cytokines and IL-6, in particular, is increased in the spinal cord during EAE [32,51], agreeing with our results in measuring *Il6* mRNA levels. By using IL6:GFAP KO, IL6:SYN1 KO, and IL6:CX3CR1 KO mice, significant decreases of *Il6* mRNA levels could be expected compared to their respective WT animals since astrocytes, neurons, and microglia, respectively, are potential sources of this cytokine; we recently described the effectiveness of these three Cre mouse models [40,48]. *Il6* mRNA levels during EAE were indeed decreased in IL6:GFAP KO and IL6:CX3CR1 KO mice, which is not surprising since astrocytes and microglia are two of the main brain cellular sources of IL-6 [3,52]. In contrast, bulk *Il6* mRNA levels of IL6:SYN1 KO mice were not different from those of their control mice. Since this model provides neuronal recombination (see [40] for further discussion), the present results suggest that during EAE neurons might not be the main cells producing IL-6 in the inflammatory cascade of this disease or maybe there exists a compensatory response by other *Il6*-expressing cells. To some extent, this can be correlated to the decrease of the clinical scores observed in the two former models, and the lack of it in the IL6:SYN1 KO mice. While systemic IL-6 KO mice are resistant to EAE [15,17], we can conclude that restricting IL-6 production solely in brain cells can eventually only reduce, but not prevent, EAE. Astrocytes seem to be the most relevant source of IL-6 in this regard. We previously showed that IL6:GFAP KO mice with limited backcrossing with C57BL/6OlaHsd mice (5 generations) had an ameliorated EAE only in females [34], which is the same result obtained in this study with floxed mice fully backcrossed (>10 generations), with a lower EAE clinical score, decreased peak and cumulative scores, and a trend for increased day to onset. In the former study, 2-month-old IL6:GFAP KO females lost less weight during EAE [34]; however, this did not occur in the present study when mice with similar ages (2–3 months old) were used, suggesting that genetic background likely influences the results in this regard. The same trends were observed in 3-5 months old microglial *Il6*-deficient mice, but they were not significant, despite *Il6* mRNA bulk levels were decreased similarly. Finally, IL6:SYN1 KO mice had a normal EAE, which in principle is consistent with their normal *Il6* mRNA levels. We cannot rule out that the wide age range of the mice (2–11 months) could influence the clinical signs, although the comparison of the younger versus the older mice yielded similar results. The body weights were altered during EAE, but in contrast to the clinical scores, no differences were observed between IL-6 genotypes and their respective WT controls. Interestingly, IL6:SYN1 KO female mice showed a lower body weight regardless of the EAE disease. It is feasible that the roles of these cellular sources of IL-6 in the control of body weight can be related to these results to some extent. Indeed, we did observe a major phenotype of IL6:SYN1 KO female mice, in line with the present results, when fed with a high-fat diet [40,48]. Taking all these data together, our results reinforce the idea that IL-6 derived from different brain cellular sources may participate in EAE pathogenesis in a cell-type specific manner.

As stated, EAE is normally considered as a peripheral disease where infiltrating T cells into the CNS have a major role [49]. However, although astrocytic (significantly) and microglial IL-6 seemed to have a role in the clinical course of EAE, no significant differences were observed in the number of CD3+ cells infiltrating the spinal cord at 19 and 27–29 dpi. Since this is a general marker of T cells, it is possible that specific T cell populations could be altered in the *Il6*-deficient animals studied. Since IL-6 acts as a key regulator in the differentiation of Th17 cells, which participate in EAE pathogenesis [21,53,54], we analyzed the presence of Th17+ cells. Indeed, IL6:CX3CR1 KO mice did show significantly decreased numbers of Th17+ cells at 27–29 dpi. Recent data support the idea of possible positive feed-back loop between IL-17 and IL-6, given that Th17 cells produce IL-17, which would interact with astrocytes and microglia, enhancing the production of IL-6 of these cells, and in turn, inducing Th17 differentiation [55,56,57].

A grossly exaggerated accumulation of activated astrocytes and microglia in the CNS are linked to EAE pathology, and it has long been known that IL-6 overexpression in the brain causes a prominent gliosis [58]. However, while there were trends for decreased gliosis, as measured by IHC, in the three models studied, the differences were small, which, together with the results already observed in Erta et al. [34], strongly suggest that astrocytes, neurons, and microglia during EAE are not critical regarding gliosis (or at least at 19, 27, and 27–29 dpi, respectively). To further determine the nature of the inflammatory response, gene expression of gliosis markers was also analyzed. Although microglial *Il6* deficiency did not have a significant effect on *Gfap* and *Mac1* expression at 27–29 dpi in comparison to their WT controls, IL6:CX3CR1 KO male mice showed lower *Gfap* expression at 15 dpi. Thus, it is feasible that in the induction phase, IL-6 is more relevant and that might be also the case with astrocytic and neuronal *Il6*, which deserves to be studied in the future. We cannot rule out, however, a SNC-specific area effect, since IHC was carried out in the spinal cord, and white and gray matter were analyzed separately, whereas qPCR was done in spinal cord (neuronal and microglial models) and medulla oblongata (astrocytic model) homogenates. We also confirmed a decreased vasogenesis in IL6:GFAP KO mice [34], which in fact is consistent with results in GFAP-IL6 mice, which display a proliferative angiopathy [58]. Since blood vessels regulate the traffic of leukocytes into the brain parenchyma, it is feasible that a decreased vascular compartment might be related to a decreased EAE. Nevertheless, this is unlikely a straightforward relationship. For instance, vasogenesis was reduced in both sexes, yet EAE was reduced only in IL6:GFAP KO female mice; and CD3+ leukocytes were not different between genotypes.

Another mechanism that contributes to EAE pathology is the recruitment of macrophages to the CNS [59]. Once microglia are activated in neuroinflammatory conditions, they acquire a macrophage-like phenotype and although recently some markers have been proposed as specific for microglia, they are still under debate. The hypothesis of polarization between classically activated macrophages/microglia (M1) and alternatively activated macrophages/microglia (M2) is hotly debated [60]. In this paper, we have focused on three markers to know whether astrocytic, neuronal, and microglial *Il6* deficiency would alter the “M1-like” (*Cd16*) and “M2-like” (*Cd206* and *Tgfb*) gene expression profile. *Cd206* expression was downregulated in microglial *Il6*-deficient males at 15 dpi. Interestingly, *Il6*-expressing macrophages M2 were associated with CD206 marker [38], demonstrating the possible positive feedback between *Il6* and *Cd206* expression. However, although *Cd206*, *Tgfb*, and *Cd16* expression were higher in MOG_35-55_-immunized mice than BSA-immunized mice at 27-29 dpi, their expressions were marginally affected by the lack of astrocytic, microglial, and neuronal IL-6. IL-6 in combination with TGFβ induces upregulation of *RORgt* expression triggering the developmental program of Th17 cells [21]. Given the lack of *Il6* expression in astrocytes, we hypothesize that the upregulation of *RORgt* mRNA levels at 19 dpi might be modulated by other factors. In fact, a previous study detailed that systemic IL-6 KO mice do not generate Th17 cells, but the deletion of Treg cells from these animals caused a reappearance of Th17 cells given the cooperation of TGFβ with IL-21 [61]. These results together with others demonstrate the complexity of IL-6 actions.

So far, the present results strongly suggest a limited role of brain IL-6 during EAE. Peripheral sources of IL-6, in contrast, are clearly important. Studies using the same floxed mice suggest that dendritic cells as well as T-cell and B-cells may be critical. Dendritic cell-derived *Il6* plays the most relevant role in EAE, since naïve T cells could not differentiate into Th17 cells because of the lack of the so-called IL-6 cluster signaling [29]. The same study also reported an amelioration of EAE in animals with *Il6* deficiency in T-cell and B-cells, although, together with a former study, the role of B-cell *Il6* deficiency may have a variable effect in MOG_35-55_-immunized mice [28,29]. In contrast, macrophage-derived *Il6* deficiency aggravated the symptoms in MOG_35-55_-immunized mice during the first days of the disease [29], revealing once again that *Il6* derived from different cells, also in the periphery, could play dissimilar role in the same context. Importantly, these results in macrophages are in sharp contrast to those observed in microglia.

## 5. Conclusions

In summary, the present results demonstrate that the symptomatology and inflammatory response to EAE may be influenced to some extent by IL-6-expressing brain cells. However, the role of IL-6 depends on the cellular source and the sex of the mice, with, in general, females being the most affected regarding astrocytic and microglial *Il6* deficiency. Interestingly, MS is more common and caused more burden in women [4], and the existence of an interplay between estrogens and IL-6 has long been known [40,46,62,63]. Clearly these sex-dependent effects must be considered and merit further studies.

## Supplementary Materials

The following are available online at https://www.mdpi.com/2073-4409/9/2/330/s1, Figure S1: qPCR primers and initial cADN concentrations. Gapdh was used as a reference gene. Figure S2: Body weight gain and clinical score. Figure S3: Representative sagittal sections of LFB/hematoxylin staining, and CD3, GFAP, IBA-1 and IL-17 immunostaining of non- immunized and MOG35-55-immunized IL6:GFAP KO mice and their respective WT controls. Figure S4: Representative images of LFB counterstained with hematoxylin staining, and CD3, GFAP, IBA-1 and IL-17 immunostaining of MOG35-55-immunized IL6:SYN1 KO mice and their controls. Figure S5: Results of FVW immunostaining of non- and MOG35-55-immunized IL6:GFAP KO mice and their controls.
